# Supplementary figures and images for: Maintenance of pure hybridogenetic water frog populations: Genotypic variability in progeny of diploid and triploid parents
Source: PLoS One. 2022 Jul 6;17(7):e0268574. doi: 10.1371/journal.pone.0268574 (PMC9258834; doi:10.1371/journal.pone.0268574)

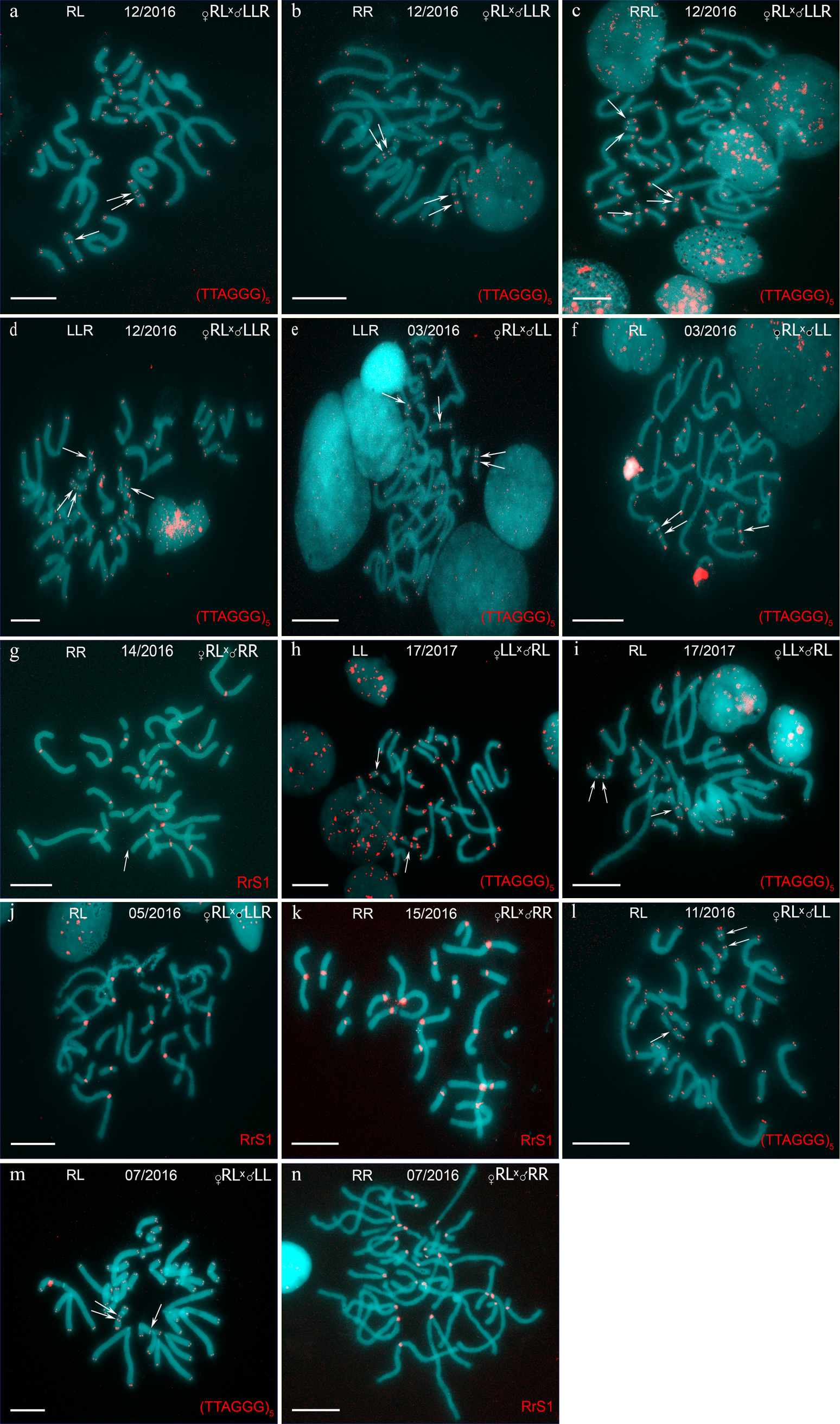

Supplement: S1 Fig — Identification of the tadpoles from crossings of diploid hybrid females with P. lessonae males (e,f,l,m), P. ridibundus males (g,k,n), diploid hybrid males (h,i), and triploid hybrid males with LLR genotype (a-d, j). Metaphase chromosomes from tadpoles after FISH with (TTAGGG)5 (a-f,h,i) and RrS1 (g,j,k,n) probes. Arrows indicate interstitial (TTAGGG)n repeat sites. According to karyotype and used species specific FISH markers we distinguished tadpoles of P. lessonae (h), P. ridibundus (b,g,k,n), diploid hybrids (a,f,i,j,l,m) and triploid hybrids with LLR (d,e) and RRL (c) genotypes. Crosses IDs correspond to Figs 1, 2 and S2 Table. Scale bars = 10 μm. (TIF) [file pone.0268574.s001.tif]

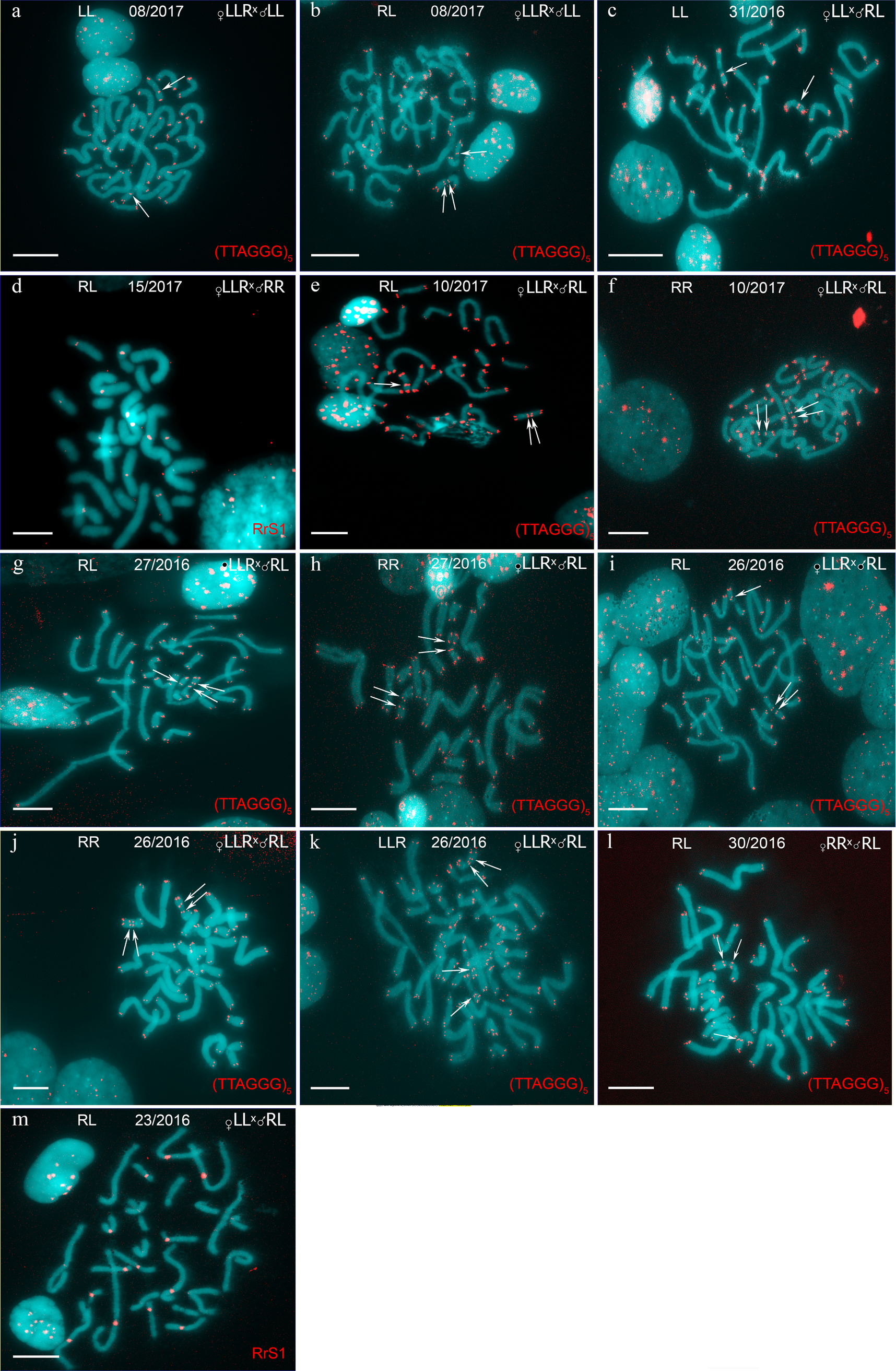

Supplement: S2 Fig — Identification of tadpoles from crossings of triploid hybrid females with P. lessonae males (a, b), P. ridibundus males (d), diploid hybrid males (e-k) as well as crosses of hybrid males with P. lessonae (c, m) and P. ridibundus (l) females. Metaphase chromosomes of tadpoles after FISH with (TTAGGG)5 (a-c,e-l) and RrS1 (d,m) probes. Arrows indicate interstitial (TTAGGG)n repeat sites. According to karyotype and used species specific FISH markers we distinguished tadpoles of P. lessonae (a,c), P. ridibundus (f,h,j), diploid hybrids (b,d,e,g,i,l,m) and triploid hybrids with LLR genotype (k). Crosses IDs correspond to Figs 1, 2 and S2 Table. Scale bars = 10 μm. (TIF) [file pone.0268574.s002.tif]
